# Comparison of novel multi-level Otsu (MO-PET) and conventional PET segmentation methods for measuring FDG metabolic tumor volume in patients with soft tissue sarcoma

**DOI:** 10.1186/s40658-017-0189-0

**Published:** 2017-09-18

**Authors:** Inki Lee, Hyung-Jun Im, Meiyappan Solaiyappan, Steve Y. Cho

**Affiliations:** 1 Radiology, University of Wisconsin School of Medicine and Public Health, Madison, WI USA; 2 Radiology, Johns Hopkins School of Medicine, Baltimore, MD USA; 3 University of Wisconsin Carbon Cancer Center, Madison, WI USA

**Keywords:** Positron emission tomography, Metabolic tumor volume, Segmentation, Soft tissue sarcoma

## Abstract

**Background:**

We have previously developed a novel and highly consistent PET segmentation algorithm using a multi-level Otsu method (MO-PET). The aim of this study was to evaluate the reliability of MO-PET compared to conventional PET segmentation methods for measuring ^18^F-FDG (FDG) PET metabolic tumor volume (MTV) in patients with soft tissue sarcoma (STS). Clinical and imaging data were obtained from the Cancer Imaging Archive. Forty-eight STS patients with FDG PET/CT and MR prior to therapy were analyzed. MTV of the tumor using MO-PET was compared to other conventional methods (absolute SUV threshold values of 2.0, 2.5, or 3.0 and percentage of tumor SUVmax values of 30, 40, 50, or 60%) and gradient-based method (PET Edge™). The reference volume was defined as an MR-based gross tumor volume (GTV). Spearman, intra-class correlation, and Bland-Altman analysis were performed to evaluate the correlation and agreement of MTV to GTV.

**Results:**

MTVs obtained using each conventional SUV parameter, PET Edge™, and MO-PET were highly correlated with the GTV in Spearman and intra-class correlation analysis (*p* < 0.05). MO-PET and PET Edge™ showed high intra-class correlation coefficient of MTV to GTV (0.93 and 0.84, respectively). The Bland-Altman bias results showed the highest agreement for MTV using MO-PET with GTV (26.0 ± 489.6 cm^3^) compared to other methods (SUV 2.0 with − 69.3 ± 765.8, 30% SUV_max_ with − 255.0 ± 876.6, and PET Edge™ with − 26.46 ± 668.82 cm^3^).

**Conclusions:**

PET MTV segmented with MO-PET showed higher correlation and agreement with GTV in comparison to conventional percentage SUV_max_ and absolute SUV threshold-based PET segmentation methods. MO-PET is comparable to PET Edge™. MO-PET is a reliable and consistent method for measuring tumor MTV.

## Background

^18^F-fluoro-2-deoxyglucose (FDG) positron emission tomography/computed tomography (PET/CT) is widely used for the initial diagnosis, restaging, and treatment response evaluation of the many kinds of tumors [1]. Among the multiple parameters that may be obtained from the FDG PET/CT, the standardized uptake value (SUV) is generally measured and accepted as an effective index [2]. In several previous studies, it was reported that tumor maximum SUV (SUV_max_) is related with the prognosis of cancers [3–5]. However, SUV has some limitations. The SUV measurement can be affected by many factors including time, blood glucose concentration, and partial volume effects [2]. SUV_max_ does not reflect the metabolic activity of the entire tumor, representing only the maximum SUV in a voxel contained within a tumor region-of-interest [6]. Also, in some tumors, the SUV_max_ is not correlated with the prognosis [7, 8]. Due to the limitations of the SUV, it is difficult to use only SUV_max_ for the prediction of tumor prognosis, and other significant PET indexes are needed. Parameters including metabolic tumor volume (MTV) and total lesion glycolysis (TLG) began to emerge compensating the role of SUV [1]. It was reported that MTV (one of the PET parameters) is related to the prognosis of various cancers [1, 3, 9, 10].

The definition of MTV, which is related to the distribution of metabolic activity, is the volume of hypermetabolic tissue that has metabolic activity exceeding a defined threshold [11]. In order to accurately measure MTV for cancer prognosis, various PET tumor segmentation methods have been attempted [12]. These various conventional methods include the absolute SUV threshold method (e.g., SUV 2.0), fixed percentage SUV_max_ threshold method (e.g., 30% SUV_max_), and signal-to-background method [9, 12]. However, the gross MTV is measured differently according to the various segmentation methods [12]. There is no standard method for measuring MTV. Therefore, among the various methods that are currently in use, the one that can best serve as a reference method remains controversial. [12].

Multi-level Otsu methods have been applied in several other application areas including in segmentation problems related to CT images. In the field of PET imaging, a variation of the basic Otsu method has been introduced as a solution to the PET segmentation problem [13]. However, in our literature search, we did not find any prior work related to the use of multi-level Otsu threshold technique applied to PET. We have applied this multi-level Otsu method to PET segmentation (MO-PET), as previously reported [14, 15]. It was demonstrated that MO-PET segmentation method is relatively accurate, stable, and consistent across a range of lesion sizes and PET lesion-to-background ratios representative of clinical tumor lesions [14, 15]. This MO-PET algorithm and method is summarized below and detailed in this reference [16] (https://www.google.com/patents/WO2016160538A1?cl=en).

Multi-level Otsu method, based on a more commonly known image threshold method known as Otsu’s method [17], is a simple and very effective clustering-based approach to convert a gray-level image to a binary image. The original Otsu method assumes that the image contains two classes of pixels (e.g., foreground and background) then calculates the optimum threshold that separates the two classes of pixels. The optimum threshold is computed such that the intra-class variance between the two classes of foreground and background pixels is minimal, which also corresponds to maximizing the inter-class variance between the two classes of the pixels. Multi-level Otsu method represents an extension of the same basic idea, i.e., minimization of the intra-class variance (which in turn results in maximization of inter-class variance) to images that contain clusters of pixel populations representing different structures that can therefore be classified at multiple threshold levels. Mathematically, MO-PET algorithm expands the original equation in the Otsu method for two pixel group classifications into an equation for classifying into an arbitrary number of classes. Thus, given the probability of occurrence of a pixel value *i* given by *P*_*i*_, the algorithm calculates the mean pixel level (*μ*) of the image and the inter-class variance (*σ*):

Mean level

$$ {\mu}_1\left({T}_1\right)=\sum_{i=1}^{T_1}i\frac{P_i}{P_1\left({T}_1\right)} $$

$$ {\mu}_2\left({T}_2\right)=\sum_{i={T}_1}^{T_2}i\frac{P_i}{P_2\left({T}_2\right)} $$

$$ {\mu}_K\left({T}_{K-1}\right)=\sum_{i={T}_{K-1}}^Li\frac{P_i}{P_K\left({T}_{K-1}\right)} $$

Variance

$$ {\sigma}_1^2\left({T}_1\right)=\sum_{i=1}^{T_1}{\left(i-{\mu}_1\left({T}_1\right)\right)}^2\frac{P_i}{P_1\left({T}_1\right)} $$

$$ {\sigma}_2^2\left({T}_2\right)=\sum_{i={T}_1}^{T_2}{\left(i-{\mu}_2\left({T}_2\right)\right)}^2\frac{P_i}{P_2\left({T}_2\right)} $$

$$ {\sigma}_K^2\left({T}_{K-1}\right)=\sum_{i={T}_{K-1}}^L{\left(i-{\mu}_K\left({T}_{K-1}\right)\right)}^2\frac{P_i}{P_K\left({T}_{K-1}\right)}, $$

where *i* is an individual SUV value (within the SUV range), *L* is the maximum SUV level in a given image, and T_1_, T_2_, … T_K-1_ are multiple threshold levels that can potentially be computed in a given image based on the distribution of the SUV within the image or a region-of-interest. Multiple threshold level values are determined by exhaustively searching through all sets of threshold levels for the given number of classes (*K*) in to which the image needs to be divided to find the combination that gives the minimum within class variance (or maximum inter-class variance). Thus, ultimately, the algorithm generates *K* classes and *K*-1 thresholds for a given image.

In this research, MO-PET, an automatic algorithm requiring very minimal user-input, was used for measuring the MTV of soft tissue sarcoma. MTVs measured with MO-PET and other conventional methods were compared in order to evaluate the usefulness and robustness of MO-PET.

## Methods

### Data acquisition

The clinical and imaging data were obtained from the Cancer Imaging Archive (TCIA: http://www.cancerimagingarchive.net), an archive of medical images of cancer through the National Cancer Institute (NCI) [18]. TCIA is an open-source and open-access database [18]. Soft tissue sarcoma database in the TCIA was used for this study [19, 20]. This dataset was acquired under a research ethics board (REB) approval by Vallières et al. [20]. A total of 51 patients with soft tissue sarcoma were analyzed.

### Image analysis

All PET/CT and MRI images were analyzed with Mirada RTx (Mirada Medical Ltd., Denver, CO, USA), with additional MO-PET segmentation algorithm developed and implemented as a plugin tool to use with ImageJ (https://imagej.nih.gov/ij/index.html), an image processing program developed by NIH. The plugin provided suitable support functions for reading and storing geometric contour information in RTSS data file format so that the contours can be exchanged between Mirada RTx and the plugin. One ellipsoidal volume of interest (VOI) containing primary tumor was drawn on each PET image. Various thresholds (absolute SUV threshold values of 2.0, 2.5, or 3.0, and fixed percentage of SUV_max_ values of 30, 40, 50, or 60%) were applied to one VOI and MTV for each threshold, termed as MTV (2.0), MTV (2.5), MTV (3.0), MTV (30%), MTV (40%), MTV (50%), or MTV (60%), respectively. MTV using MO-PET software (MTV (MO-PET)) was obtained using the identical VOI which was applied to the various threshold methods. For the reference standard volume, the tumor contours defined on the MRI were used. The MR contours, which were previously manually drawn on T2-weighted fat-suppression (T2FS) scans by Villiers et al., were obtained from the TCIA database [19, 20]. The gross MR-based tumor volume (GTV) was measured on the MR images with Mirada RTx using obtained MR contours. MTV was also measured with PET Edge™ (MIM software Inc., Cleveland, OH, USA), a gradient-based PET segmentation method. The ratio of each MTV using various thresholds to the GTV was calculated in order to evaluate the accuracy of each MTV segmentation method. The closer the ratio of GTV to each MTV is to 1, the MTV is regarded as a better segmentation method compared to GTV.

### Statistical methods

Data are expressed as mean ± SD. Spearman correlation, intra-class correlation coefficient, and Bland-Altman analysis were used to compare the data of MTVs obtained with various thresholds and MO-PET. Each volume was compared to that of MRI-derived GTV. Data were evaluated using statistics software (Medcalc version 10.1.7.0, Medcalc software, Mariakere, Belgium).

## Results

### Patients

Fifty-one soft tissue sarcoma cases were obtained from the TCIA [20]. Among them, three cases were excluded as measuring the MTV was inappropriate. In one case, the tumor was located at the left upper arm adjacent to the PET/CT gantry, which made it impossible to draw a precise VOI due to its location. The tumors of the other two cases had large edema around the primary tumor. The huge discrepancy between tumor and edema precluded accurate tumor delineation. After ruling out the 3 cases, a total of 48 cases were included for the final analysis. The features of tumor including histology, location, grade, SUV_max_, and GTV are summarized in Table 1. Detailed clinical data can be accessed via the TCIA site (10.7937/K9/TCIA.2015.7GO2GSKS).

**Table 1:** Tumor characteristics

Information	Number (%) (*n* = 48)	Range of SUV_max_ (mean)	Range of GTV (cm^3^) (mean)
Histology			
Liposarcoma	10 (20.8%)	3.4015.94 (7.73)	111.17–1532.36 (561.66)
Malignant fibrous histocytomas	17 (35.4%)	5.37–28.19 (14.97)	17.17–2336.46 (632.67)
Leiomyosarcoma	9 (18.8%)	3.98–29.99 (13.36)	54.18–420.72 (227.38)
Synovial sarcoma	5 (10.4%)	6.78–28.71 (13.78)	35.11–543.73 (262.59)
Fibrosarcoma	1 (2.1%)	5.39	79.22
Extraskeletal bone sarcoma	3 (6.3%)	7.80–27.77 (22.68)	206.06–743.95 (403.11)
Other	3 (6.3%)	9.18–17.46 (12.30)	79.90–727.70 (402.13)
Location of primary tumor			
Pelvis	8 (16.7%)		
Thigh	29 (60.4%)		
Calf	5 (10.4%)		
Knee	2 (4.2%)		
Upper extremity	4 (8.3%)		
Grade			
High	26 (54.2%)		
Intermediate	15 (31.3%)		
Lower	4 (8.3%)		
Ungraded	3 (6.3%)		

### Tumor volume

The ratio of MTVs using each threshold and MO-PET to gross MR-based tumor volume (GTV) was calculated. MO-PET and the gradient-based method showed MTV to GTV ratio close to 1, at 1.12 ± 0.42 and 1.08 ± 0.38, respectively (Table 2). These ratios were most significant on Spearman correlation and intra-class correlation analyses. Percentage SUV_max_ and absolute SUV threshold method-based PET segmentation did not perform well in comparison to GTV. Among the fixed percentage SUV_max_ threshold methods, (30% SUV_max_, 40% SUV_max_, 50% SUV_max_, and 60% SUV_max_) MTV (30%) showed the highest ratio of MTV to GTV (0.64 ± 0.35), while MTV (2.0) showed the highest ratio of MTV to the GTV (1.00 ± 0.61) among the absolute SUV threshold methods (SUV 2.0, SUV 2.5, and SUV 3.0).

**Table 2:** Correlation between PET MTVs and MRI GTV

MTV parameter	Ratio of MTV to GTV	Spearman correlation coefficient	Intra-class correlation coefficient (95% CI)
MO-PET	1.12 ± 0.42	0.945***	0.93 (0.88–0.96)
PET Edge™	1.08 ± 0.38	0.947***	0.84 (0.71–0.91)
SUV 2.0	1.00 ± 0.61	0.799***	0.79 (0.62–0.88)
SUV 2.5	0.80 ± 0.55	0.680***	0.73 (0.52–0.85)
SUV 3.0	0.65 ± 0.50	0.561***	0.68 (0.44–0.82)
30% SUV_max_	0.64 ± 0.35	0.738***	0.42 (− 0.04–0.67)
40% SUV_max_	0.39 ± 0.28	0.621***	0.21 (− 0.40–0.56)
50% SUV_max_	0.23 ± 0.20	0.426**	0.10 (− 0.60–0.50)
60% SUV_max_	0.12 ± 0.13	0.291*	0.04 (− 0.71–0.46)

*Significant at *p* = 0.045; **significant at *p* = 0.003; ***significant at *p* < 0.001

**Fig. 1:**
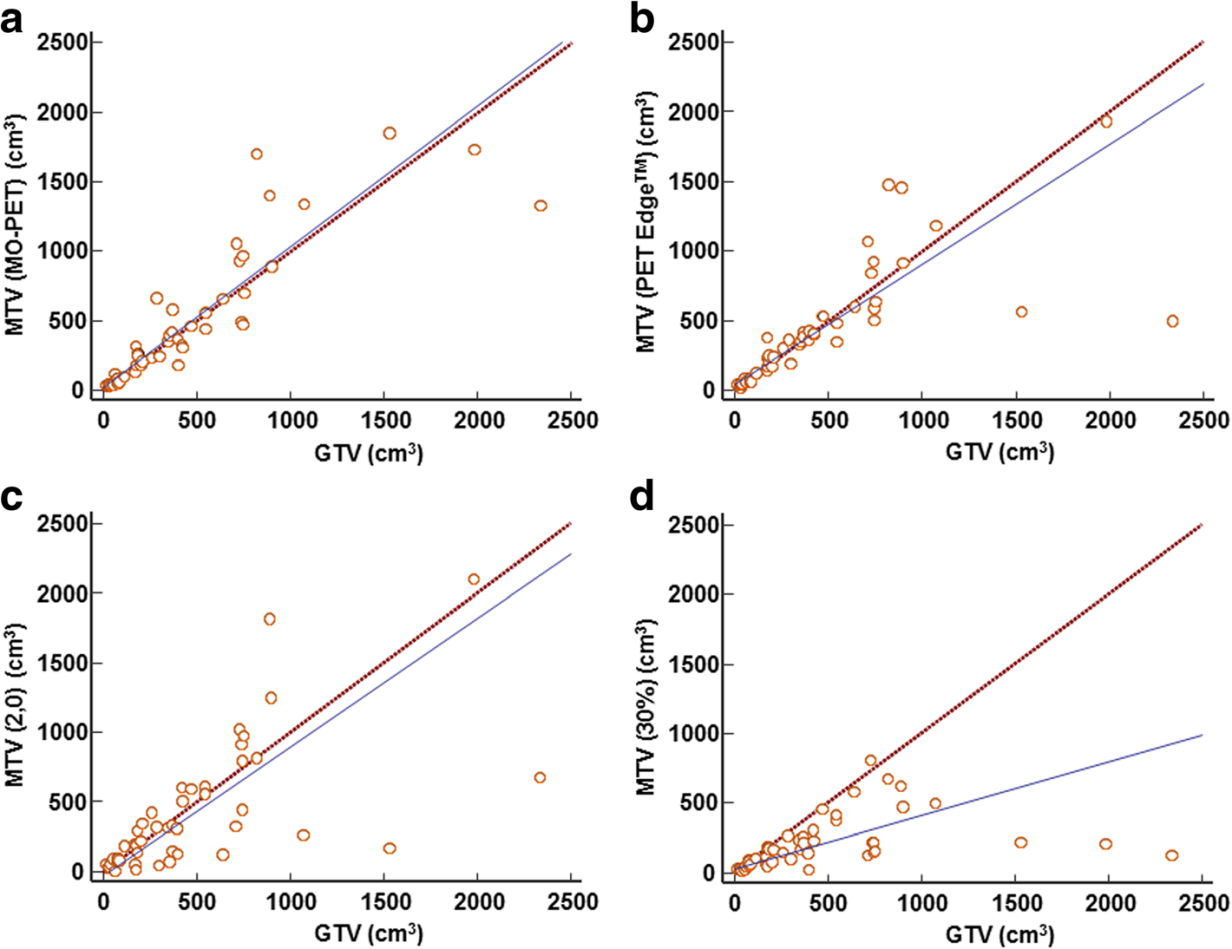
Spearman correlation for MTV and GTV. **a** MO-PET. **b** PET Edge™. **c** Absolute SUV threshold value of 2.0. **d** Percentage of SUV_max_ value of 30%. A significant positive correlation is exhibited between each MTV and GTV. Solid line, trend line; dotted line, line of equality

MTV with MO-PET and the gradient-based method demonstrated similar high correlation with GTV (Spearman correlation coefficient; 0.945 and 0.947, respectively; Table 2). Percentage SUV_max_ and absolute SUV threshold method-based PET segmentation did not perform as well in comparison. Spearman correlation coefficients (*r*) of MTVs using 30% SUV_max_, 40% SUV_max_, 50% SUV_max_, 60% SUV_max_, SUV 2.0, SUV 2.5, and SUV 3.0 to the reference GTV were 0.738, 0.621, 0.426, 0.291, 0.799, 0.680, and 0.561, respectively (50% SUV_max_, *p* = 0.003; 60% SUV_max_, *p* = 0.045; all other parameters, *p* < 0.001; Table 2). In the correlation graph, each MTV measured by various methods and GTV showed a significant correlation with each other (Fig. 1). Furthermore, MTV (MO-PET) exhibited the most accurate trend line with GTV compared with those of other MTVs.

MO-PET showed the highest intra-class correlation coefficient compared to the reference GTV with 0.93 (95% CI, 0.88–0.96; Table 2), slightly better than the gradient-based method with 0.84 (95% CI, 0.71–0.9; Table 2), although the correlations in both the methods were not statistically significant.

**Fig. 2:**
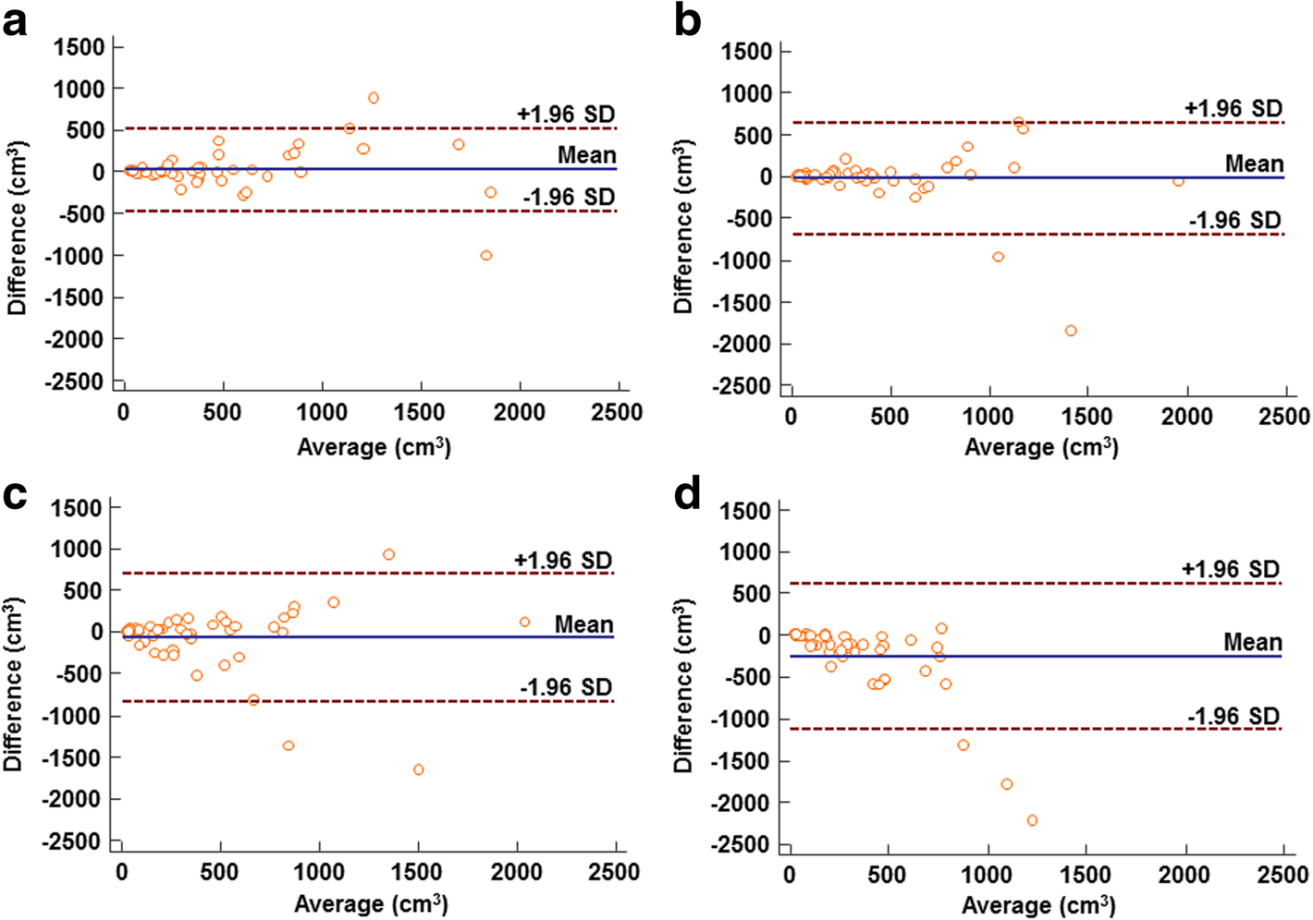
Bland-Altman analyses for MTV and GTV. **a** MO-PET. **b** PET Edge™. **c** Absolute SUV threshold value of 2.0. **d** Percentage of SUV_max_ value of 30%. The Bland-Altman bias results show 26.0 ± 489.6, − 26.46 ± 668.82, − 69.3 ± 765.8, and − 255.0 ± 876.6 cm^3^ for MO-PET, PET Edge™, SUV 2.0 and 30% SUV_max_, respectively

The Bland-Altman analysis showed biases between each MTV and reference GTV, with the lowest variation for MO-PET. The biases of MTV with MO-PET, 30% SUV_max_, SUV 2.0, and PET Edge™ were 26.0 ± 489.6, −55.0 ± 876.6, −69.3 ± 765.8, and −26.46 ± 668.82 cm^3^, respectively (Fig. 2).

**Fig. 3:**
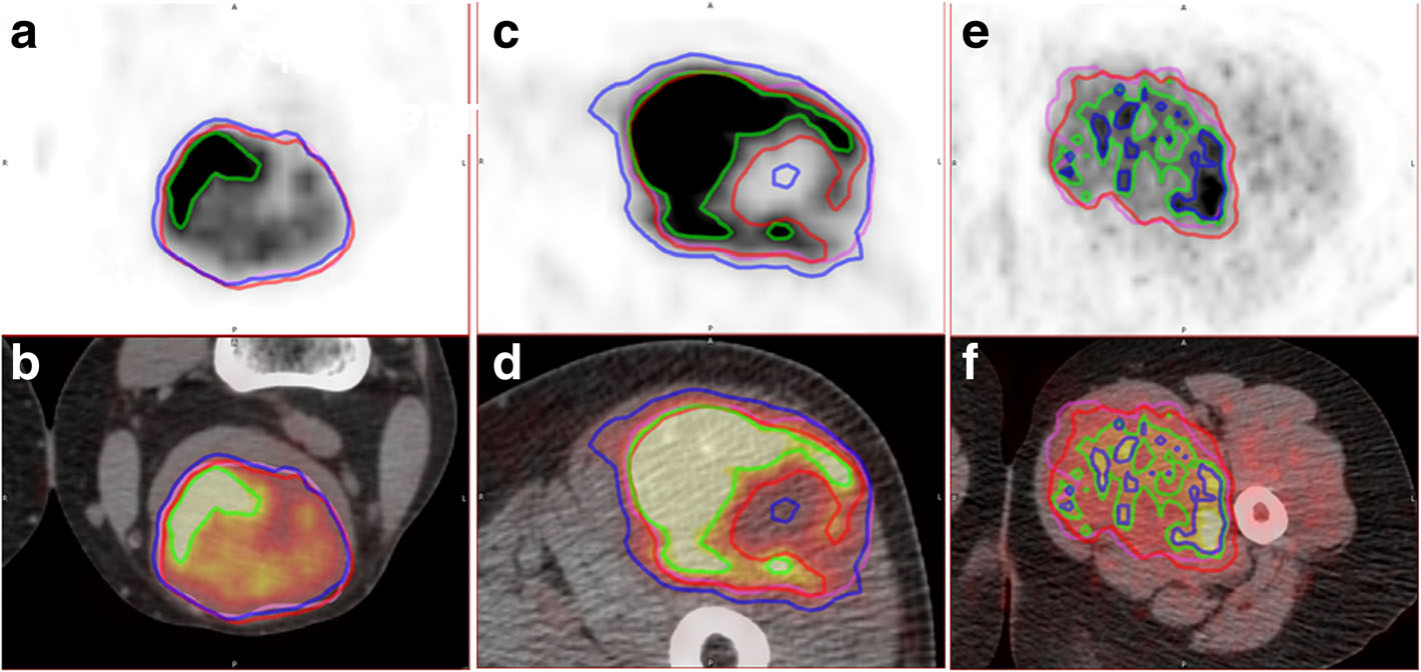
Images of representative cases (**a, c,** and **e** axial ^18^F-FDG PET image; **b, d,** and **f** axial fusion image). **a, b** A 54-year-old male with synovival sarcoma in the left popliteal fossa (SUV_max_ 28.71). The ratios of MTV to GTV for MO-PET, SUV 2.0, 30% SUV_max_, and PET Edge™ are 1.05, 1.06, 0.25, and 0.97, respectively. **c, d** A 64-year-old male with extraskeletal osteosarcoma in the right thigh (SUV_max_ 27.77). The ratios of MTV to GTV for MO-PET, SUV 2.0, 30% SUV_max_, and PET Edge™ are 0.90, 1.62, 0.57, and 1.17, respectively. **e, f** A 42-year-old female with pleomorphic leiomyosarcoma in the left thigh (SUV_max_ 5.47). The ratios of MTV to GTV for MO-PET, SUV 2.0, 30% SUV_max_, and PET Edge™ are 1.13, 0.24, 0.46, and 0.99, respectively. Red line, MO-PET; blue line, SUV 2.0; green line, 30% SUV_max_; pink line, PET Edge™

Images of representative cases are shown in Fig. 3.

## Discussion

In this study, we evaluated the usefulness of the newly developed PET segmentation method, MO-PET, for measuring MTV. MTVs measured using MO-PET and various threshold methods were compared to the MRI-derived GTV obtained from the TCIA database. It was demonstrated that MO-PET and the gradient-based method (PET Edge™) showed comparable MTV, with the highest correlation to GTV. In addition, these two methods were superior to the absolute SUV and percentage SUV_max_ threshold methods.

As shown in the result, the calculated ratio of MTV (2.0) to GTV was most close to 1. However, the SD of MTV (MO-PET) ratio was smaller than that of MTV (2.0). Furthermore, the Spearman and intra-class correlation coefficients of MTV (MO-PET) were higher than that of MTV (2.0). According to the Bland-Altman analysis, the MTV (MO-PET) and MR-based tumor volume showed superior agreement to other methods.

The Bland-Altman analysis showed that MTV(MO-PET) had strong correlation regardless of the tumor volume, while the tumor volume measured using absolute SUV threshold (SUV 2.0) or fixed percentage SUV_max_ threshold (30% SUV_max_) showed greater discrepancy as the tumor volume increased. Furthermore, the absolute SUV threshold methods showed some limitation of tumor delineation, in cases where there were heterogeneous metabolic activities in the tumor [21]. In such instances, the fractional parts of the tumor with metabolic activity lower than the threshold could get excluded from MTV measurement, and this may result in underestimation of the metabolic tumor volume [21]. On the other hand, MO-PET showed good tumor delineation in cases where tumor had heterogeneous SUV. In the case of tumor with the high SUV_max_, fixed percentage SUV_max_ threshold method may show underestimated MTV. Also, the MTV of the tumor which has low SUV_max_ may be undervalued with the absolute SUV threshold method. It was reported that the underestimation of MTV in the patients with low SUV_max_ would be possible [22]. The MO-PET algorithm may solve this problem.

In order to further evaluate MO-PET against commercially available software, PET Edge™ of MIM software was used to measure MTV. PET Edge™ measures MTV based on the gradient-based segmentation method [23]. Spearman correlation coefficients of MO-PET and PET Edge™ showed relatively similar values. In terms of intra-class correlation coefficient, MO-PET was slightly higher compared to that of PET Edge™. Segmentation using MO-PET was a comparable method to the gradient-based segmentation method. However, MO-PET derives the tumor contour with simple VOI, while the gradient-based segmentation method requires manual adjustment for the tumor contour. As a result, reproducibility of tumor contour using the gradient-based method may show inconsistency if the tumor is irregular in shape or has much necrotic portion in the tumor.

Recently, MTVs are increasingly studied for the prediction of the prognosis of various cancers [7, 8, 10, 11]. Superior correlation between MTV and tumor prognosis is also reported compared to that of the SUV_max_ [24]. However, there is no ideal method established for the measuring MTV [12]. It is difficult to predict the prognosis with MTV that is measured with the non-established, various threshold methods. There are many conventional methods including absolute SUV threshold method, percentage SUV_max_ threshold method, lesion-to-background method, and gradient method for tumor segmentation which are used to measure MTV. MTVs depend on various threshold methods [11, 12, 21]. Manually drawn segmentation method can also be used on the MRI or CT with visual assessment; however, tumor volume in this method can be affected by how the segmentation is drawn [25]. Therefore, the development of reproducible and automatic tumor segmentation method is needed. The MO-PET method was developed in order to overcome these limitations. We previously evaluated that MO-PET is relatively accurate, stable, and consistent for measuring MTV using standard NEMA image quality phantom study compared to conventional threshold methods [14]. In addition, it is evaluated that MO-PET can be applied to the clinical images in this study.

Regarding soft tissue sarcoma that was analyzed in this study, several researches on the correlation between the PET parameters and tumor prognosis have been reported. However, the results reported have been conflicting with each other. For instance, it was reported in one study that there is positive correlation between the PET parameters (including SUV and other volume parameters) and metastasis [20]. In another, it was also reported that SUV_max_ and other volume parameters including MTV and TLG are related to tumor prognosis [3]. Whereas, Hong et al. reported that volume-based parameters are not correlated with tumor prognosis, but only SUV_max_ is correlated with disease progression [26]. Also, it was reported that TLG is a superior prognostic index to SUV_max_ and MTV [27]. Due to these contradictions and discrepancies, it is necessary to study the correlation between MTV and tumor prognosis with optimal MTV measurements.

There were two limitations in this study: (1) the reference standard volume was defined using the tumor contour on the MRI as an appropriate anatomic comparator for PET MTV; but MRI tumor contours are not necessarily a definitive reference standard. Also, there may be discrepancies between the volumes using the MRI contour with the actual pathologic volume; (2) the PET/CT images that were analyzed in this study do not have whole body images. So, lesion-to-background method cannot be compared in this study.

## Conclusions

In conclusion, PET MTV segmented with MO-PET method showed higher correlation and agreement with MRI-based GTV in comparison to conventional percentage SUV_max_ threshold and absolute SUV threshold-based PET segmentation methods. MO-PET is a reliable and consistent method for measuring tumor MTV. Quantitation of tumor metabolic burden using the MO-PET segmentation method shows very good assurance by its results for future clinical applications.
